# Using artificial neural networks to predict future dryland responses to human and climate disturbances

**DOI:** 10.1038/s41598-019-40429-5

**Published:** 2019-03-07

**Authors:** C. E. Buckland, R. M. Bailey, D. S. G. Thomas

**Affiliations:** 1 School of Geography and the Environment, University of Oxford, Oxford, OX1 3QY UK; 2 Geography, Archaeology and Environmental Studies, University of the Witwatersrand, Johannesburg, South Africa

## Abstract

Land degradation and sediment remobilisation in dryland environments is considered to be a significant global environmental problem. Given the potential for currently stabilised dune systems to reactivate under climate change and increased anthropogenic pressures, identifying the role of external disturbances in driving geomorphic response is vitally important. We developed a novel approach, using artificial neural networks (ANNs) applied to time series of historical reactivation-deposition events from the Nebraska Sandhills, to determine the relationship between historic periods of sand deposition in semi-arid grasslands and external climatic conditions, land use pressures and wildfire occurrence. We show that both vegetation growth and sediment re-deposition episodes can be accurately estimated. Sensitivity testing of individual factors shows that localised forcings (overgrazing and wildfire) have a statistically significant impact when the climate is held at present-day conditions. However, the dominant effect is climate-induced drought. Our approach has great potential for estimating future landscape sensitivity to climate and land use scenarios across a wide range of potentially fragile dryland environments.

## Introduction

## Landscape Reactivation in Dryland Systems

Land degradation and surface sediment remobilisation are major issues in drylands^1^, which cover 40% of the earth’s land surface^2^, with their consequences estimated to directly affect up to 2 billion people^3^. Surface destabilisation induces a variety of problems for local communities^2,4,5^ including the removal of fertile soils and nutrients^6^, the burial of small plants, and damage to infrastructure, livestock and crops^7^. The interactions between climatic stress (i.e. drought)^8^, land use^9–11^, grazing pressure^12–16^ and wildfire^17–21^ bring about damage to surface protective vegetation cover^22–24^ and expose the underlying sediment to erosion by aeolian processes and land degradation^4^. Disturbance-driven degradation can lead to the reactivation of dryland geomorphological features, such as sand dunes, resulting in the formation of localised blowouts^24–29^ or even large-scale dune remobilisation in some of the world’s most vulnerable regions^30,31^. In turn, the creation of blowouts acts as an initial source of sediment that can lead to further reactivation and new dune formation^25^.

With increases in population pressure and land use, and climate models predicting increasing precipitation variability^32^, there is a greater need to understand the sensitivity of dryland landscapes to future disturbances. In particular, there is a specific concern about how *individual* forcing factors interact with the environment to induce instability and land degradation^22^. Current methods of analysis often rely on simple visual comparisons between historic records and the correlation of individual forcing parameters against periods of reactivation in the record^27,33^, or short-lived individual event analyses in the study of aeolian system surface change^34^. Univariate analyses focus on individual drivers (see review^27^) but fail to identify the feedbacks between the range of climatic and anthropogenic forcing factors. At the Quaternary timescale sand dune reactivation studies, for example, have long been used as a proxy for palaeoaridity^35–37^, with luminescence-derived chronologies used to infer periods of historic drought^33,38–40^. Whilst a focus on climatic factors may be suitable on millennial timescales, more recent localised reactivations are driven by a combination of natural and anthropogenic disturbances. Aside from a climatic focus, many studies have commented on the role of wildfire^17–21^ or grazing pressures^12–16^, which in themselves demonstrate key biogenic relationships, but might mask the synergies within the wider system. Whilst some studies have suggested the combined role of multiple forcing factors in contributing towards vegetation removal and landscape destabilisation^16,22,41–50^, few have quantified the relationship between forcing factor and environmental response using traditional analytical methods.

Quantifying the role of individual parameters and identifying the complex relationship between the forces and geomorphological response is therefore essential to predicting the likelihood of future disturbance-driven reactivations. Ideally, a process-based model would be used to simulate hypothetical geomorphic responses to future climatic and anthropogenic forces. However, these mechanistic models do not yet exist in the form necessary for dryland aeolian environments.

Here, we apply a ‘data-led’ empirical approach, rather than trying to construct a mechanistic model (that comes with the problems of structural and parameter uncertainty). We explore the potential for novel applications of artificial intelligence (AI) techniques to improve our understanding of the relationship between climatic and anthropogenic disturbances and resultant geomorphic response in dryland environments. Artificial neural networks (ANNs) provide a hitherto underutilized method to simultaneously examine multiple forcing factors and uncover their complex relationships^51^. A supervised learning algorithm takes a known set of paired input-target data and trains a model which can be used to generate reasonable predictions for the response to new unseen data. As such, ANNs have been applied to a range of research problems from predicting stock markets^52^ to biomedical sciences^51^ and monitoring water quality^53^ to name a few. Specifically in environmental research, ANNs have successfully been applied in a range of dendroclimatic^54–57^, geomorphological^58,59^ and aeolian studies^60–63^.

## Experimental Design

The goal of this study is to identify how changes in drivers of vegetation disturbance induce changes in sediment mobilisation. The main drivers of vegetation disturbance and subsequent sediment reactivation are: climate-induced drought, grazing/land use pressure and wildfire disturbance; both currently and expected in the future. In this study, two ANNs were established to predict the likelihood for identifying episodes of sediment deposition in near-surface sand dune profiles given a set of forcing factors (i.e. climate, grazing pressure and wildfire occurrence). Luminescence-dated sediments from near-surface dune sands provide a historical chronology of deposition events which are used to represent episodes of sediment mobility within the system over the past 400 years (see Methods). Ideally, continuous time series of relevant climate data would be used as an input to train the first ANN (ANN1), and this is indeed possible over more recent time periods. However, such records are not long enough to cover the time period represented by the sediment deposition dates (c. the last 400 yrs). After searching for longer-term records of relevant data, and proxies for climate conditions, we found tree ring growth indices to provide the best available data. Rather than train the ANNs against climate data directly, we therefore trained them against tree ring data as a proxy for climate^54,64–66^. In addition, we used historical records of grazing pressure and wildfire occurrence (see Methods and Supplementary Note 2).

Using empirical data, we first identify the relationship between driving forces and sediment deposition events from the historical record before varying the disturbance factors under a set of future scenarios (alternate climatic, grazing and fire frequency futures) to deduce the environmental response and sensitivity to individual parameters. As the ANN was trained using historical tree ring data, future climate scenarios must then also be expressed in this way. A second ANN predicted hypothetical tree ring growth under two different future climatic regimes (ANN2; see Methods). We used two hypothetical climate futures combined with varying wildfire regimes and grazing pressures to sensitivity test the likelihood of deposition events when forcing factors were modelled under a range of conditions. All details are provided in Methods and in the online Supplementary Material.

## Nebraska Sandhills: a test case

With the experimental design defined, this approach was applied to empirical data collected from the Niobrara Valley Preserve in northern Nebraska, US. Empirical data from a detailed analysis of historical (10^1^–10^3^ years) land degradation in the Nebraska Sandhills (Buckland *et al*. submitted 16/08/18), a region synonymous with the 20th century US Dust Bowl and with the potential to experience serious disturbance in future decades^67^, provides input to this analysis. For our modelling experiments, we chose chronologies of depositional events from six different geomorphological features in the northern Nebraska Sandhills to provide a measure of environmental response to disturbance events over the last 400 years (see Supplementary Note 1). Six separate profiles, from a range of geomorphological features within the local area, are used for inter-site comparison (see Methods and Supplementary Note 3). Input datasets taken from a tree ring growth index (a proxy for climate), a simplified measure of historical grazing/land use pressure, and tree scar records (a proxy for wildfire history) provide the inputs for the model, representing natural and human disturbance forces (see Supplementary Note 2).

ANN1 defines the relationship between climatic and human disturbance forces and the resultant episodes of near-surface sediment deposition. Luminescence-dated periods of deposition are presented as a continuous estimate of probability based on multiple discrete dates spanning the period of interest. ANN2 identifies the historical relationship between climatic inputs (annual growing season precipitation, average max and min temperatures) and tree ring growth^65^ which is used for the second stage of sensitivity testing.

Model validation and testing were achieved through two methods: (i) partitioning of the original dataset, and (ii) cross-validation between the dune sites. Datasets were randomly partitioned for training, validating and testing purposes during the training of the network. ANN1 consists of an input layer, four hidden layers (comprising 3:30:30:3 fully connected neurons) and an output layer, whilst ANN2 has an input layer, one hidden layer (9 neurons) and an output layer. ANN1 is a time-delay network, incorporating a delay line of up to 8 years between datasets for input (forcings) and output (sediment age probability) (see Methods).

## Results

### Model training

**Figure 1:**
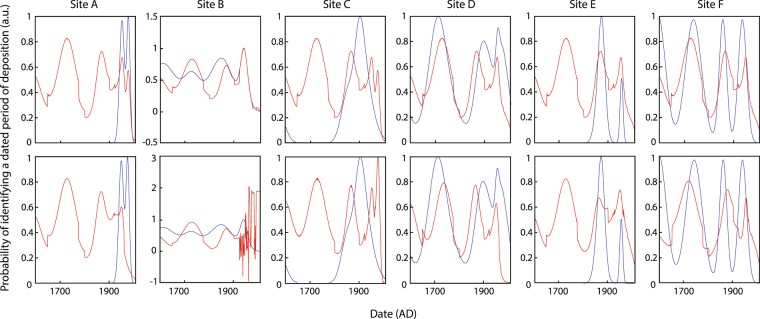
Results from both in-sample (*upper*) and out-of-sample (*lower*) testing across the six sites in ANN1. Blue lines refer to the known target datasets taken from empirical measurements of periods of deposition, and red lines are the predicted model outputs for each respective site. Due to the incomplete nature of sedimentary profiles^75^, periods with no evidence of depositional events have been removed from the training dataset to prevent the model identifying these conditions as conducive to stability (i.e. sites A, C and E).

For each of the six study sites, when all sites were included in the training dataset (Fig. 1: *upper*, *‘in-sample’ prediction*), the timings of predicted deposition events (red) align well with the known dataset reconstructed from historical sediments (blue). Additional simulated peaks in the record (centred on 1730 and 1870 AD) suggest episodes of sediment deposition that we would expect to find if deeper sediment samples were extracted at sites A, C and E.

Cross-validation results (‘*out-of-sample*’ prediction) demonstrate the capacity to accurately predict the six known depositional profiles when the respective site has been excluded from the network training stage (Fig. 1: *lower*). All sites, except B, show good correlation between the peak positions of the known target dataset and predicted model output.

### Scenario testing

**Figure 2:**
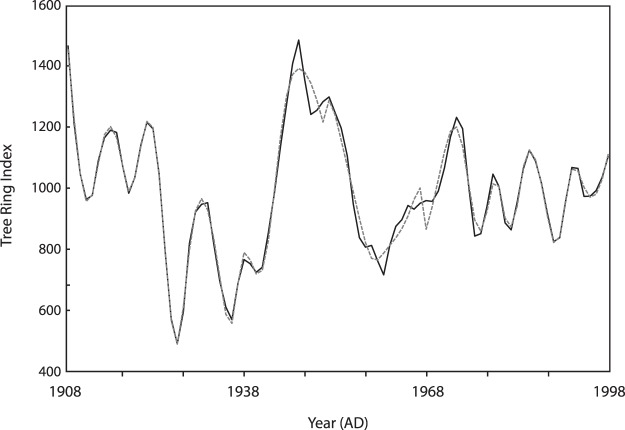
Results from training network ANN2 simulating the relationship between growing season precipitation and temperature conditions with tree ring growth over the period 1908–1997 AD. Black line represents known historical tree ring growth index^65^ (smoothed 0.1 RLOESS), grey line is simulated tree ring growth based on the trained network.

**Figure 3:**
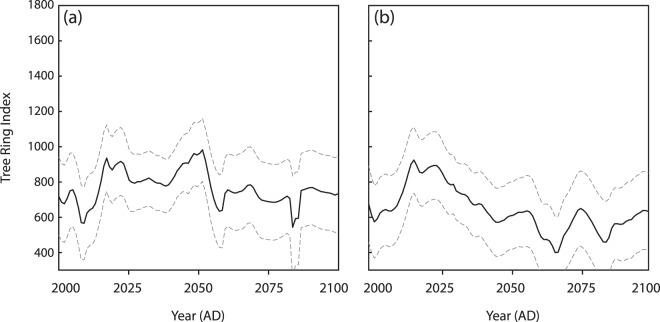
Simulated tree ring growth indices based on two different climate scenarios: (**a**) Climate 1 (long-term averages), (**b**) Climate 2 (low emission conditions with gradually increasing temperatures (see Methods). Mean (black) and standard errors (grey dashed) were calculated using 102 repeats. Multiple repeats ensured that the simulated dataset was not driven by random extreme values.

Having effectively trained the model, in this section we assess how example combinations of the key drivers (climate, grazing pressure and wildfire) are expected to induce changes in sediment mobility, given the relationships learnt from historical data. First, using ANN2, after successfully predicting historical tree ring growth from historical growing season precipitation totals and average max and min daily temperatures (Fig. 2) (see Supplementary Note 4), two new tree ring growth futures were simulated based on two hypothetical climates (climate 1 & 2) (see Methods and Supplementary Note 4) (Fig. 3). Climate 1 growing season precipitation totals and max/min temperatures follow the trend set by long-term averages (1908–2015 AD). For comparison, climate 2 growing season temperatures increase by 4.5 °F by the end of the century based on future regional projections across a suite of climate models under a low emission scenario^68^.

Tree ring growth outputs across both climatic profiles produced results in line with theoretical expectations. After 8,000 model repeats, datasets which produced multiple extreme values outside of the bounds of the training set were excluded (see Methods); the mean and standard error were calculated with the remaining repeat datasets (Fig. 3). Climate 1 shows a noisy signal with low-level sensitivity to precipitation and temperature changes (Fig. 3a) while the greater climatic stresses of climate 2 force a reduction in tree ring growth with time (Fig. 3b).

**Figure 4:**
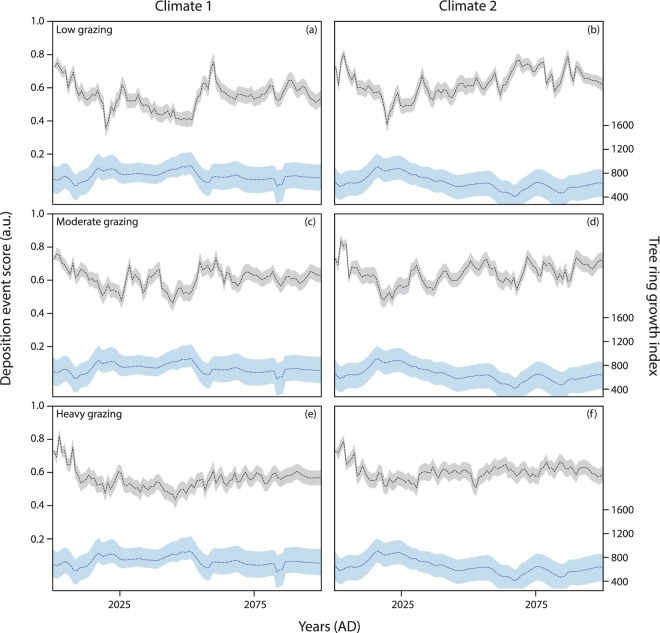
ANN1 and ANN2 simulated output results based on two future climate scenarios and three different grazing pressures. Blue output refers to simulated tree ring growth index based on climate 1 and 2 hypothetical scenarios. Grey output demonstrates ANN1 likelihood of identifying a depositional event (ANN1 output is plotted as ‘deposition event score’ where relative high points correspond with an increased likelihood of identifying an episode of deposition in the sediment). Dotted lines depict mean values calculated after repeat runs were screened for optimum model performance, shaded area corresponds with standard error. Repeat runs were screened according to ability to predict the correct location of deposition events in the training dataset (see Methods).

Second, using the trained ANN1 and three levels of grazing pressure, simulated tree ring growth indices produced six future likelihoods for identifying deposition events in the near-surface sediments. Across both climate futures, an antiphase relationship between tree ring growth and the likelihood for sediment deposition (deposition event score) suggests sediment movement is largely driven by climatic conditions (Fig. 4, see Supplementary Note 6). Under historical average climatic conditions (climate 1) a noisy signal is seen across all three combinations of grazing conditions with the output profile in general mirroring the signal found in the tree ring growth index (Fig. 4a,c,e). Increased aridity (in climate 2), reduced the tree ring growth index with time, and similarly increased the likelihood of identifying a period of deposition in the sediment (i.e. higher deposition event score) (Fig. 4b,d,f). This relationship is particularly visible under low grazing conditions but is less well-defined with noisy shallow peaks under higher levels of grazing pressure.

**Figure 5:**
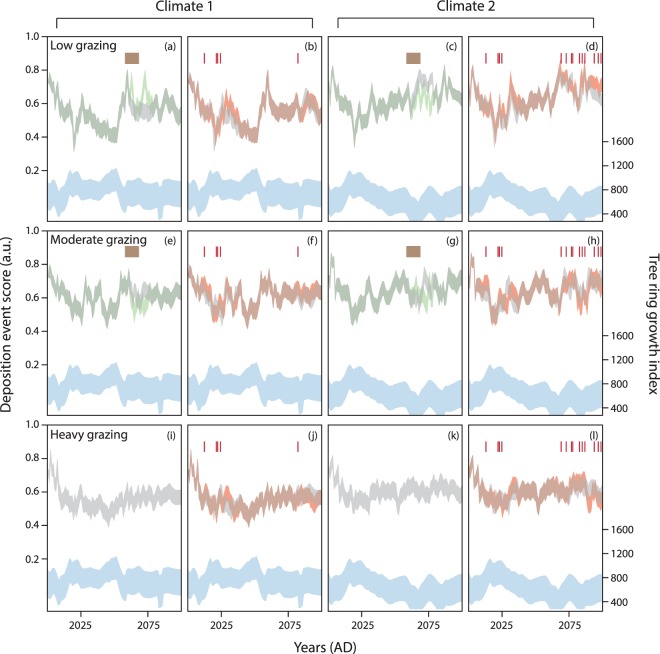
Output results when new scenarios of climatic conditions, grazing pressure and wildfire occurrence are combined over a 100-year period. Deposition event scores are presented as mean ± standard error following 5,000 repeats. Repeat runs were screened according to ability to predict the correct location of deposition events in the training dataset (see Methods). Six different disturbance scenarios (see Methods) are plotted across the 4 columns for each of the 3 different grazing pressure levels (Low grazing: **a–d**, Moderate grazing: **e–h**, Heavy grazing: **i–l**). Column 1: compares scenarios 1 and 2. Column 2: compares scenarios 1 and 3. Column 3: compares scenarios 4 and 5. Column 4: compares scenarios 4 and 6. Blue output: simulated tree ring growth index based on either climate 1or 2. Grey output: ANN1 OSL output based on climate-only scenarios. Green output: includes added grazing pressure scenarios. Brown bar depicts period of increased grazing pressure. Red output: includes wildfire. Red bars represent years with wildfire occurrence.

Third, to explore the added impact of increased grazing pressure (above background levels) and wildfire frequency, additional grazing pressure was added to low and moderate grazing regimes for a 10-year period (Fig. 5 brown bars) and wildfires were added when average max temperatures >80 °F (Fig. 5 red bars). Temperature is considered a strong determinant of fire frequency in the Great Plains^69^. Under low grazing conditions, an increase in pressure results in a lagged-system response with a notable increase in the likelihood to identify episodes of sediment deposition (Fig. 5a). By contrast, when background grazing conditions are already at moderate levels, or when climates are tending towards more arid conditions, an increase in grazing pressure does not alter the model projections (Fig. 5c,e,g); the signal is driven by climatic disturbances as demonstrated in the tree ring growth index. Additional grazing pressure was not added to the already heavily grazed scenarios (Fig. 5i–l).

A complex relationship with wildfire occurrence shows a marginal increase in the deposition event score following a wildfire event. Typically, wildfire occurrence results in an amplified likelihood of sediment deposition 1–2 years after the event and tends to follow the general trend of the output profile defined by the climatic conditions. An increased amplification of the sediment deposition likelihood associated with wildfire is particularly apparent under low grazing conditions with consecutive wildfires (Fig. 5b,d). Under heavily grazed conditions, wildfire events do not cause a statistical increase in the likelihood of identifying sediment mobility in the record, except for following a series of three wildfire events (Fig. 5j: 2021–2024 AD) under average climatic conditions (climate 1).

## Discussion

### Drivers of future deposition events

Results highlight the dominance of climatic disturbance in driving vegetation growth (as shown in the tree ring growth index) which in turn explains the majority of the variability found in the deposition event score (ANN1 output profile). When additional grazing (or land use pressure) is added to a previously low pressure grazed environment, a significant increase in identifying periods of sediment deposition is found. Yet, in environments under already stressed conditions (i.e. moderate/heavy grazing or drier climates), an increase in pressure bares no additional impact on the likelihood of identifying sediment movement in the geomorphic system. These results agree with previous studies^70,71^ that have commented on the non-linear nature of dryland geomorphic systems, whereby a threshold of disturbance is required to initiate sediment reactivation. Our results show that the force exerted by external disturbances is not linearly related to the likelihood for periods of sediment deposition to be identified in the record; the geomorphic response is binary not continuous. Additional grazing pressure does not increase the likelihood for reactivation in a system that is already experiencing sediment mobilisation, but it might contribute to pockets of localised heterogeneity in the record and, in particular, the formation of blowouts^29,72^.

Whilst some studies have suggested that fire may not contribute to the likelihood of sediment reactivation in semi-arid grassland environments^17^, our findings support existing research^18,20,21^ that has suggested that wildfires may damage surface vegetation and increase the likelihood for sediment remobilisation (e.g. Fig. 5d: 2083–88 AD period) and during periods of low climatic stress (Fig. 5b). Under low and moderate grazing regimes, model outputs predict a marginal increase in the deposition event score following the 2012 wildfire. In 2012, a large wildfire (76,000 acres) stripped the study region of trees, shrubs and grass species. Yet, despite the extensive nature of the fire, anecdotally ranchers and preserve managers commented on the lack of evidence for sediment remobilisation following this event – likely caused by extensive root networks^17^, sediment crusting^73^, and wildfire management practices^74^. Changes in grazing strategy and landscape management following wildfires have been instrumental in determining the subsequent likelihood for sediment remobilisation. The model used in this example has been trained on the land management practices during the 20^th^-century and is unable to predict how future human decision-making will change. As such, whilst the model predicted a sediment response post-2012 fire, the lack of physical evidence to support this simulation is likely caused by non-analogous behaviour to the training dataset.

### Limitations of the approach

Whilst our results have demonstrated the potential application of ANNs for estimating future landscape sensitivity, it is essential to equally understand the limitations of the method used. Out-of-sample predicting is the main limitation for the approach outlined here; this is the likely inability to provide accurate forecasts when models are trained on a limited or non-analogous dataset. The unique set of input conditions found at site B is not replicated at any of the other 5 sites, and without another set of comparable data to train from, the predicted output is beyond the limited conditions represented in the training dataset (e.g. Fig. 1 site B). In this study, we have attempted to reduce such errors in expanding the dataset by training across multiple study sites. As in all inferential methods, a larger dataset that encompasses more drought cycles would improve the training (and therefore the predictable capabilities) further. An inherent weakness of data-informed models in this context is that under future climate change, if forcing conditions (or combinations of conditions) stray significantly far from past experience (significantly ‘non-analogous’), supervised models may no longer be able to accurately predict future landscape responses.

Another difficulty using this method is associated with identifying what the relative peaks and troughs in the simulated outputs represent. As mentioned earlier, the network has been trained on a luminescence-dated target dataset which provides a continuous estimate of the probability of identifying a luminescence age (i.e. episode of deposition) in the sedimentary record. However, ascertaining what peaks in the simulation output correspond with a deposition ‘event’ in the profile is dependent on where the relative baseline associated with stability is positioned (i.e. are all periods with scores >0 indicative of sediment mobilisation episodes?). In this study, the network has been trained on definitively identified episodes of sediment deposition, not periods of known stability (see Methods). A series of erosional filters gradually remove evidence of historic reactivation events from the sedimentary system^75^. As such, our target dataset does not provide evidence for all deposition events, but more importantly, it does not provide definitive evidence of periods of stability. There is a distinct difference between an absence of evidence and an evidence of absence, but this is not resolved in the depositional record. A baseline at zero therefore, to represent no likelihood of identifying a deposition event in the forecasted plots, is incorrect and thus events are inferred as the most prominent relative peaks in the deposition event profile as opposed to those that are identified above a set threshold, or those that are representative of extreme model runs (see Supplementary Note 5).

**Figure 6:**
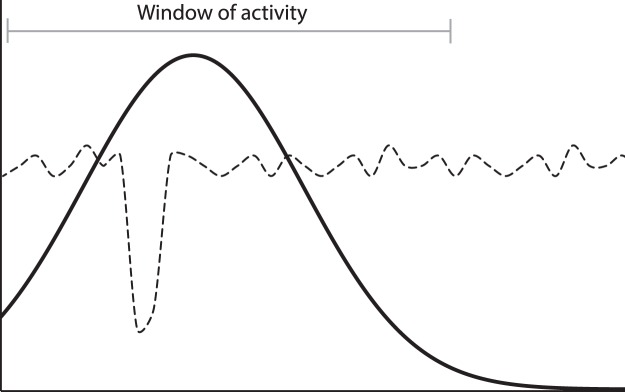
Schematic figure demonstrating how the wide training window of activity as defined by the target dataset (solid line) forces the model to train the equivalent period of input datasets (dashed line) to that target – even if the true ‘disturbance’ was only a short-lived event.

The relatively large error margins associated with dated episodes of deposition (see Supplementary Note 3) further compound the difficulty in identifying specific deposition events and associated conditions. This is problematic for the network training as input conditions over large windows are considered to be inducing a short-lived sediment reactivation event that is characterised by large uncertainties (Fig. 6). Conditions that are therefore reflective of landscape stability may falsely be associated with identified periods of deposition. Since the model has trained against a dataset that has already undergone a series of natural filters^75^, the simulated outcome does not necessarily depict every deposition event. Additional known point-in-time observations taken from historical aerial imagery, oral records, agricultural reports would improve the resolution of the training.

### Wider significance

We have shown the capacity to use ANN models coupled with empirical datasets to improve our understanding of aeolian geomorphological systems and future sensitivity to climatic and human disturbance. Our results demonstrate how ANNs can identify relationships between paired historical input-target datasets, as well as the potential for trained models to make predictions about the likelihood of future reactivations. When known forcings are included in the simulations, we can identify peaks in reactivation driven largely by perturbations in climatic conditions, but also grazing pressure and fire frequency when aridity stresses are low. Results from this study suggest that fire can increase the capacity for near-surface reactivation and could be used to explain heterogeneity in local sediment profiles under low-stress climatic conditions. Likewise, the resilience of grasslands to grazing pressures is largely related to the ambient climate^71^. As expected, the dynamic nature of dryland environments means that the likelihood for sediment reactivation is not linearly related to individual forcings. Through ANN simulations, we demonstrate the dynamic relationships between disturbances and response, exploring the idea of thresholds and a lagged-response to external perturbations.

The black box nature of ANNs prevent a defined weighting of each disturbance parameter being produced, but sensitivity testing has demonstrated the non-linear response of the system to different combinations of disturbance conditions. These findings corroborate with existing research that synergies between forcing parameters are the key to near-surface reactivations in semi-arid grasslands^43,45^.

Using cross-validation tests, we have demonstrated the bounds within which these models most optimally perform. Without all potential outcomes represented in the original training dataset, the ability to predict accurate outcomes is limited. If future models are to accurately predict future sediment reactivation events, non-linear multivariate tools are needed to fully-integrate the variety of parameters influencing near-surface activation. ANNs represent an opportunity within drylands, and wider landscape dynamics, to define environmental relationships and thresholds in systems where process-based mechanistic models are absent. The potential for future models, however, to accurately simulate hypothetical futures is constrained by the capacity to identify suitable training datasets that capture analogues for forthcoming conditions.

## Methods

All neural networks have been developed in Matlab R2017b using the Neural Network toolbox and code written by the authors (CEB & RMB).

### ANN1

ANN1 is an artificial neural network used to identify the relationship between climatic and anthropogenic disturbances and the likelihood of a luminescence age, indicating sediment deposition, to be found in the sedimentary record. ANN1 was trained with input-target data from 1590–1997 AD and used to stimulate the likelihood of identifying future deposition events in the sediment in the following 100 years under a series of alternative climatic and grazing pressure scenarios (see Future scenario forecasting).

#### Pre-processing input datasets

Three primary contributors to vegetation cover, and thus surface stability, were selected for inclusion in the model: grazing pressure, wildfire occurrence, and climatic conditions. Instrumental measurements (capturing the last 100 years of climatic data) were not used due to the relatively short time series measured. A longer dendrochronological record of tree ring growth from the Niobrara Valley Preserve (NVP)^65^ provided an integrated record of precipitation and temperature change over the past 400 years. Annual tree ring growth data was extracted and smoothed by a factor of 0.2 RLOESS function, to allow a signal to be identified by the model amongst the environmental noise. Grazing pressure for the six dune sites was based on the levels identified in the index defined in Buckland *et al*. (submitted – 16/08/2018) and smoothed by a moving average factor of 0.1 to represent progressive changes in management strategy. Wildfire occurrence was included as a binary input (i.e. ‘1’ – fire, ‘0’ – no fire) based on the fire scar records from NVP^76^.

#### Pre-processing target dataset

The target dataset for each of the six dune sites was represented by a probability density function (PDF) of stacked luminescence (optically stimulated luminescence – OSL) ages that depict episodes of sediment deposition in the dune profile. The target dataset represents a likelihood of identifying an OSL age, it does not necessarily capture every sediment reactivation event that has occurred for two reasons. First, historical events may have been eroded from the sedimentary record, and whilst a multicore strategy has been used to increase the overall representation of reactivation events, due to the fragmented nature of aeolian sedimentary profiles some events may be eroded from the record. Second, the event may not have been captured in the OSL dating due to sampling strategy. Considering these caveats, whilst a peak in the PDF dataset represents a depositional event, a trough does not equally represent a period of stability. A positive peak is a true result, but a negative absence only suggests that we did not find evidence of an event. As such, it is essential to pre-process the target dataset to avoid falsely training the ANN to interpret certain conditions as conducive to stability. Where long gaps between peaks (regions of contiguous values at zero) were found in the luminescence record, target values were removed, preventing the model from associating periods which might be due to erosional loss of sediment to specific environmental conditions, thereby learning a false relationship (for the present purposes).

#### Model structure and architecture

**Figure 7:**
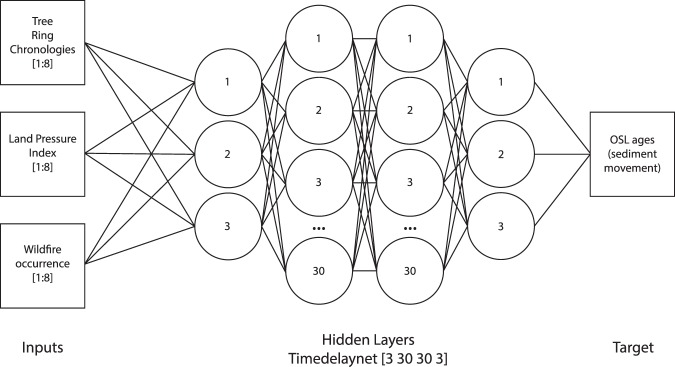
Schematic diagram demonstrating the model architecture of the final models used in ANN1. Training model was based on input-target dataset from 1590–1997 AD, with scenario datasets based on measured climatic data from 1998–2014 and simulated tree ring growth indices from 2015–2098.

Final model structure used ‘timedelaynet’ function to allow for an adjustable lag to be incorporated into the network architecture. A lag of up to 8 years for the input variables was selected following a series of trials of different time lags. Given the complex nature of the relationship between the input variables and the resultant likelihood of sediment movement, four hidden layers were used with a structure of [3 30 30 3] neurons in the layers (Fig. 7). The Levenberg-Marquadt algorithm was used to train the network (minimizing distance between model outputs and target data) and the mean square error (MSE) of each model run was used as the performance indicator for each iteration. Training datasets were randomly partitioned for training (90%), validation (5%) and testing (5%) purposes during cross-validation experiments, but to reduce over-fitting in the future scenario simulations this split was altered to increase the test portion of the datasets (50% training, 5% validation, 45% testing) and improve the robustness of the model outputs.

### ANN2

ANN2 is an artificial neural network used to identify the relationship between individual climatic parameters (growing season precipitation, average max and min daily temperatures) and tree ring growth index^65^ from 1908–1997 AD. ANN2 was used to produce simulated tree ring growth indices under a range of hypothetical new climatic scenarios.

#### Pre-processing datasets

Precipitation and temperatures measurements (sourced from Ainsworth Met Station (42.58°N, −100.05°W)) were used as input datasets and smoothed by a factor of 0.2. A tree ring growth index produced using ponderosa pine tree rings within the NVP was used as the target dataset^65^.

#### Model structure and architecture

**Figure 8:**
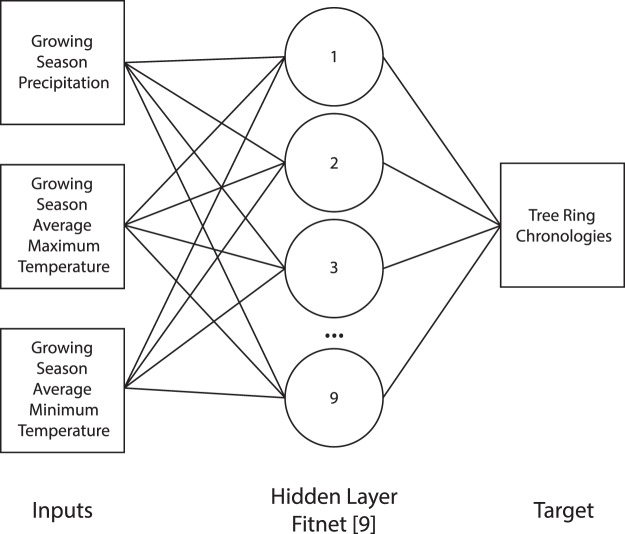
Schematic diagram demonstrating the model architecture of the final models used in ANN2. Training model was based on input-target dataset from 1908–1997 AD, with scenario datasets based on measured data from 1998–2014 and simulated data from 2015–2098.

Final model structure used ‘fitnet’ function, a single hidden layer with 9 neurons (Fig. 8) and the Levenberg-Marquadt algorithm. Known time series data was randomly partitioned for training (70%), validation (10%) and testing (20%) purposes.

### Future scenario forecasting

Future scenario data refers to the 100 years post-1998 with the period from 1998–2014 using measured instrumental data, followed by 84 years of future ‘scenario’ data based on a range of climatic conditions, grazing pressure and wildfire frequency.

#### Scenario Forecasting (part 1): Tree Ring Growth Index

Trained ANN2 was used to model the predicted tree ring growth indices associated with two hypothetical climate scenarios and used as the new inputs alongside combinations of grazing pressure and wildfire occurrence in ANN1. Future climate data (2015–2100 AD) was produced using an autoregressive model to identify the normal levels of ‘noise’ identified in the measured historical precipitation and temperature datasets, before being added to two different future trends (Table 1) (see Supplementary Note 4).

**Table 1:** Description of two different climate scenarios used to generate forecasted tree ring growth indices.

	Growing season precipitation total (inches)	Growing season average maximum & minimum observed temperatures (°F)
1	Growing season precipitation based on long-term average (last 100 years) ± noise generated from auto-regressive model of long-term precipitation trends.	Growing season average maximum and minimum temperatures based on long-term average (last 100 years) ± noise generated from auto-regressive model of long-term temperature trends.
2	Growing season precipitation based on long-term average (last 100 years) ± noise generated from auto-regressive model of long-term precipitation trends.	Growing season average maximum and minimum temperatures based on long-term average (last 100 years) plus low emission projections of a 4.5 °F increase in temperatures by the end of the century ± noise generated from auto-regressive model of long-term temperature trends.

Two combinations of future growing season precipitation, average maximum temperatures and average minimum temperatures have been used to produce different climatic conditions. Scenarios used do not demonstrate predicted future conditions but are used as proof on concept to demonstrate the capacity of the model under a range of forcing combinations. Climate 2 temperature settings are based on projections of the Central Great Plains region under a low emissions scenario^68^. Regional climate summaries were based on IPCC models and the findings of the NCA Report (2014).

Using the training dataset (1908–1997 AD) to define ANN2, the model was ran for 8,000 repeats with the simulated outputs associated with each network repeat stored by the model. Multiple cycles of the network ensured that the simulated target dataset was not driven by random extreme values. Repeats that produced more than seven tree ring indices >5,000 (i.e. extreme values) were excluded from the final dataset. The mean and standard error for each year was calculated for the two climatic scenarios and used as the new tree ring growth index for simulating future sediment deposition events in ANN1.

#### Scenario Forecasting (part 2): Future likelihood of sediment deposition events

Simulated tree ring indices were modelled against three levels of grazing pressure: low, moderate and heavy. Further analysis to sensitivity test the role of wildfires and additional periods of grazing pressure when applied against a background level were completed to produce six different combinations of future disturbance conditions (Table 2).

**Table 2:** Description of six combinations of future climatic, land use and wildfire conditions that have been used in this study to simulate alternate sediment movement likelihood predictions across the six sites studied.

	Tree ring (climate) scenario	Land use pressure	Wildfire frequency
1	Climate 1 (Long-term averages)	Current land pressure index values used for all sites.	No wildfires.
2	Climate 1 (Long-term averages)	Current land pressure used, except increase by factor of 1 for 10-year period from year 2059	No wildfires.
3	Climate 1 (Long-term averages)	Current land pressure index values used for all sites.	Wildfire in years with average maximum temperature >80 °F.
4	Climate 2 (Low emission)	Current land pressure index values used for all sites.	No wildfires.
5	Climate 2 (Low emission)	Current land pressure used, except increase by factor of 1 for 10-year period from year 2059	No wildfires.
6	Climate 2 (Low emission)	Current land pressure index values used for all sites.	Wildfire in years with average maximum temperature >80 °F.

Using the known datasets (1590–1997 AD) to define ANN1, 5,000 repeats were completed using the six training sites. The training dataset was partitioned with a relatively low training portion (50%) and high validation and testing partition (5% + 45%) (based on a short time series) to prevent over-fitting and improve the robustness of the future scenario outputs. With each repeat, the model simulated the predicted future likelihood of identifying depositional episodes in the sediment, classified as a deposition event score, according to the six future disturbance scenarios.

#### Model validation and selection

**Figure 9:**
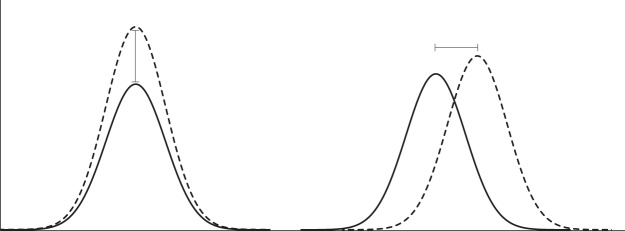
Schematic diagram demonstrating that MSE caused by variation in magnitude of peaks between training and predicted datasets (left) is not useful because the event occurred at the same point in time. The difference in the location of the peak (right) in time is a more appropriate measure of model performance.

Repeats were assessed according to the model’s ability to correctly reproduce the peaks found in the known historical deposition dataset (ANN1 training target). ANN1 training target is characterised by a continuous curve with peaks of varying magnitude, but the nature of the response is binary (i.e. sediment moves, or it does not). The size and magnitude of the model-derived peaks are therefore not significant, with the location of the peaks relative to the known dataset (Fig. 9) a good indication of overall model performance and ‘fit’. Given this, the mean squared error (MSE) of the model is not an appropriate method for determining overall model performance. In this study, we assessed the performance of the model based on the variation in location between the known and modelled peaks, with the best model identified as having the closest alignment of peaks across the six study sites. To identify the optimum models, we sum the differences between training and predicted peak positions for each model repeat across the six sites and select the models of best fit. The model identified a predicted peak which fell within a defined time window of the target peak and calculated the difference in location between the two peaks. If a target peak was not reproduced in the predicted dataset (i.e. a predicted peak does not occur within the peak window), a model penalty value (e.g. 1e6 used in this study) was assigned. Total peak location differences and penalties were summed across the identified peaks in each site, and across the six sites for each neural network repeat run. A moderate number of iterations (300) per repeat was used to prevent the model from attempting to improve the network to the optimum MSE, which is not necessarily the best peak-location-performing model. Maximum peak window size was defined as half the distance between the two closest known peaks in the target dataset (i.e. 13 in this study).

Following 5,000 repeats, the mean and standard error associated with the repeats from the top 10% of models was calculated (model repeats for each timepoint were normally distributed). Increasing the percentage of models used to calculate the mean and standard error confidence intervals would reduce the overall uncertainty on the profile, but potentially includes the output from models that are poorly trained against the known datasets and are therefore more precise but less accurate (see Supplementary Note 5).

### Code Availability

The model is implemented in MatLab^®^ (version R2017b) using code written by the authors. A full version of the neural network code is freely available on GitHub (https://github.com/CatherineBuckland/ANN).

## Supplementary information

Supplementary Information

## Data Availability

The authors declare that most of the data supporting the findings of this research are within the paper and corresponding Supplementary Information. Any other additional data supporting the results presented are available from the corresponding author upon request.
