# Supplementary material for: Using artificial neural networks to predict future dryland responses to human and climate disturbances
Source: Sci Rep. 2019 Mar 7;9:3855. doi: 10.1038/s41598-019-40429-5 (PMC6405911; doi:10.1038/s41598-019-40429-5)
Supplement: Supplementary file 1 — Supplementary Information [file 41598_2019_40429_MOESM1_ESM.docx]

**Supplementary Information:**

**Using artificial neural networks to predict future dryland responses to human and climate disturbances**

Catherine E. Buckland^1^, Richard M. Bailey^1^, David S G. Thomas^1^

^1^School of Geography and the Environment, Oxford University Centre for the Environment, South Parks Road, Oxford, OX1 3QY, UK

**NOTES**

1. **Study sites**

Study sites used in this paper are part of a wider project investigating the drivers of near-surface reactivation in the northern Nebraska Sandhills, US. Six sites in the vicinity of the Niobrara Valley Preserve (NVP) and surrounding ranches were sampled for near-surface dune sediments and periods of surface deposition were dated using luminescence (optically stimulated luminescence – OSL) methods (Buckland et al. submitted – 16/08/2018) (Figure 10). Luminescence ages date the period that has elapsed since the sediment was last exposed to sunlight, as such providing a date of deposition in the landscape.


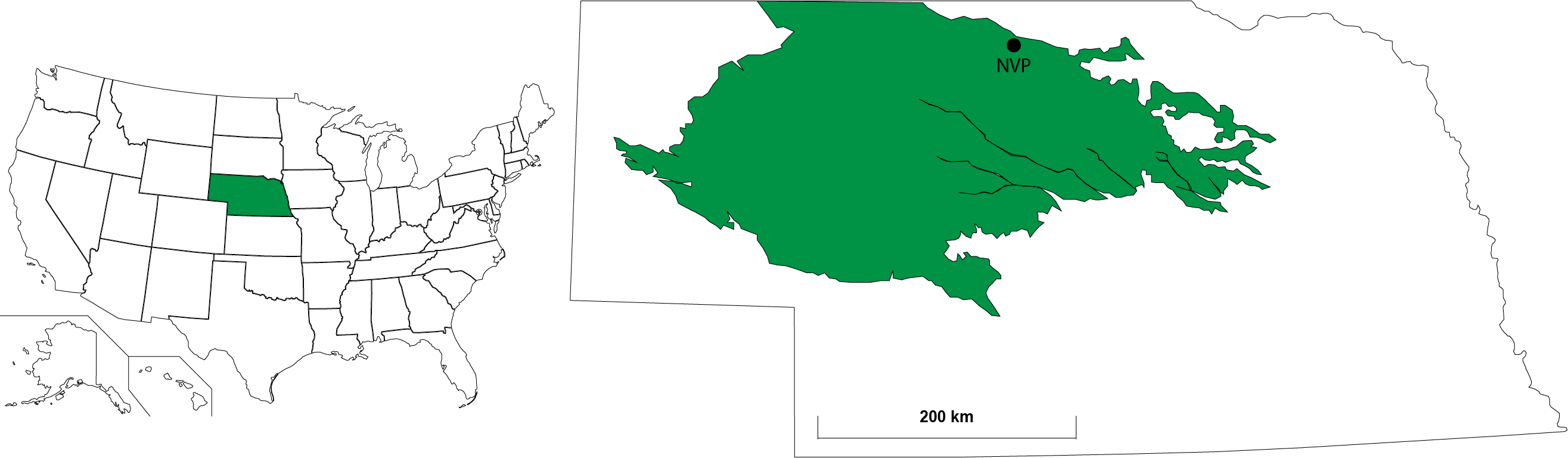


**Figure 10.** Location map of Niobrara Valley Preserve (NVP) within the Nebraska Sandhills (green inset) and United States. Six study sites were measured for depositional history in the NVP and surrounding ranches. Please see Buckland et al. submitted – 16/08/2018 for further details on the geomorphic setting of individual study sites.

1. **ANN1 Inputs**

**
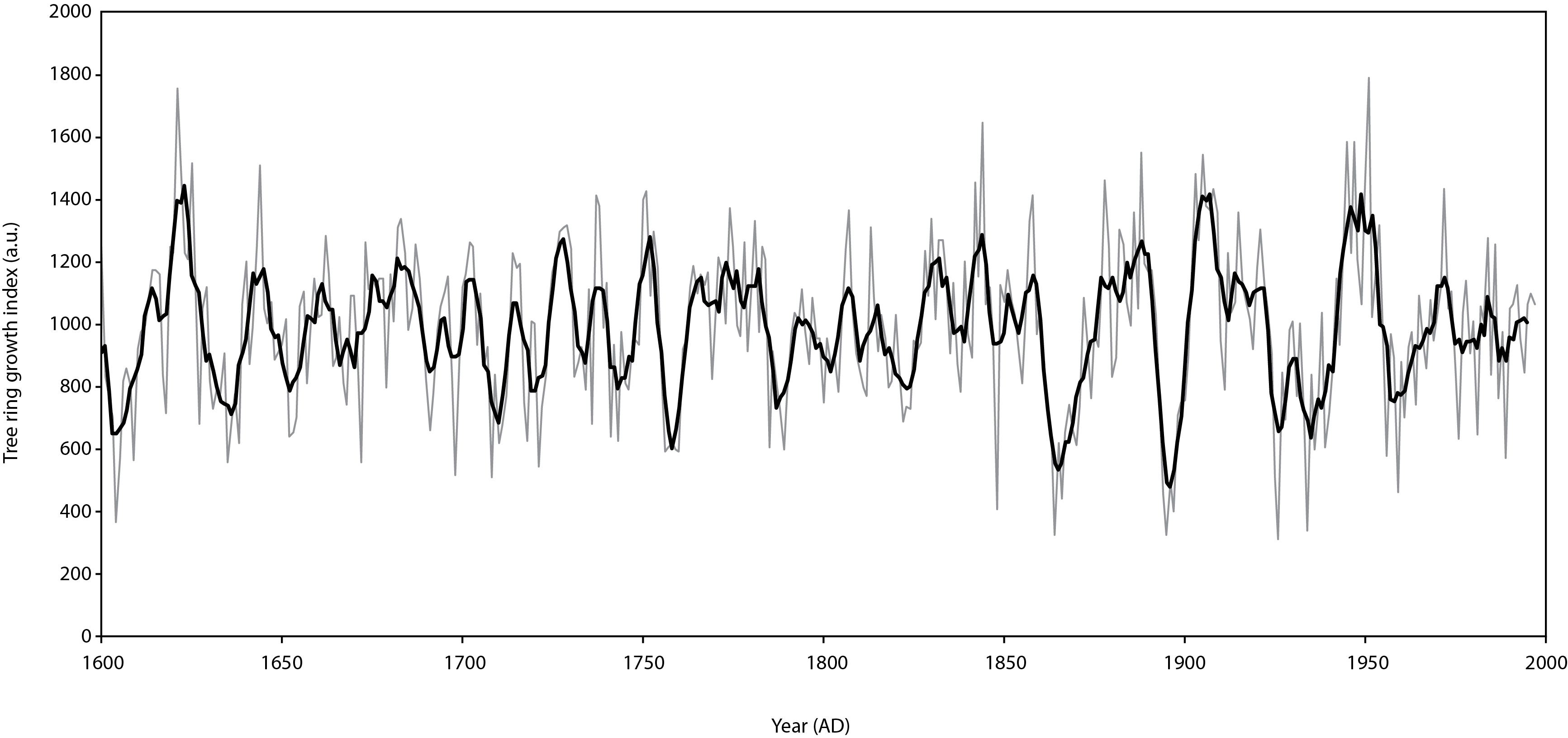
Tree ring index (also used as ANN2 target) from 1590-1997:** A local tree ring growth index ^1^ produced using samples of Pinus ponderosa was used as a proxy for historical climatic conditions over the last 400 years (Figure 11).

**Figure 11.** Tree ring growth index compiled using multiple ring width from ponderosa pines in the vicinity of the NVP. Data extracted from: Brown et al. ^1^ Grey line depicts annual tree ring growth index values, whilst the black line shows a 5-year rolling average (illustrated here to demonstrate natural peaks and troughs in the record and the association with climatic episodes – e.g. note the troughs associated with the widely reported droughts of the 1890s, 1930s and 1950s).

**Grazing pressure:** Annual grazing (or land use) relative pressure on the landscape was defined by a three-point scale reflecting the changes in relative grazing pressure on the local sampling sites over the past 400 years (Table 3). Without access to historical stocking rates and animal movements over this period, a three-point scale of relative pressure was used to depict broad-scale changes in the levels of pressure exerted on the land at each of the six sites over time. Refer to Buckland et al. (submitted – 16/08/2018) for further details on the construction of this index.

**Table 3.** Summary table of the grazing pressure exerted on each of the six sites over the last 400 years. A three-point scale of relative pressure was used to capture the combination of population dynamics, grazing and agricultural practices, land ownership chronologies and wild grazing versus commercial ranching. Time brackets and intensity scores have been discussed and ratified with local grassland ecologist Dr Al Steuter. Classification scores reflect: 1 – low pressure, 2 – moderate grazing pressure, 3 – high grazing pressure.

| **Period** | **Site and Intensity score** |
| --- | --- |
| Pre-1870s | All sites: 2 |
| 1870-1880 | All sites: 1 |
| 1880-1890 | All sites: 1 |
| 1890-1904 | Sites A, B, C & D: 2  Sites E & F: 3 |
| 1904-1930 | All sites: 3 |
| 1930-1940 | Sites A, C, D, E & F: 2  Site B: 3 |
| 1940-1970 | Sites A, C, D, E & F: 2  Site B: 3 |
| 1970-1980 | Sites A, B & C: 3  Sites D, E & F: 2 |
| 1980s onwards | Sites A & C: 1  Site B: 3  Sites D, E & F: 2 |

**Wildfire record:** A history of wildfire occurrence in the local area was extracted from a local tree scar record (collected by Professor T Bragg – University of Nebraska-Omaha) ^2^. A binary input was used to reference years when a wildfire did (‘1’) and did not (‘0’) occur with no pre-processing of the dataset required.

1. **ANN1 Target: OSL PDF datasets**

The target dataset used in ANN1 is a stacked chronology of the identified periods of sediment deposition at six sites within the Niobrara Valley Preserve and surrounding ranches. At each of the six dune sites, a series of OSL dates were calculated to reconstruct a history of episodes of sediment deposition that had occurred in the recent history across the different sampling sites (Buckland et al. submitted 16/08/2018). The target dataset used in this study represents a likelihood of identifying a luminescence age (referring to a period of deposition) in the sampled sediment, it does not necessarily capture every sediment deposition event that has occurred, but only those that have not been eroded from the sequence ^3^.

The final luminescence ages associated were stacked in a probability density function (Figure 12) for each of the six sites to produce the target dataset used in ANN1.


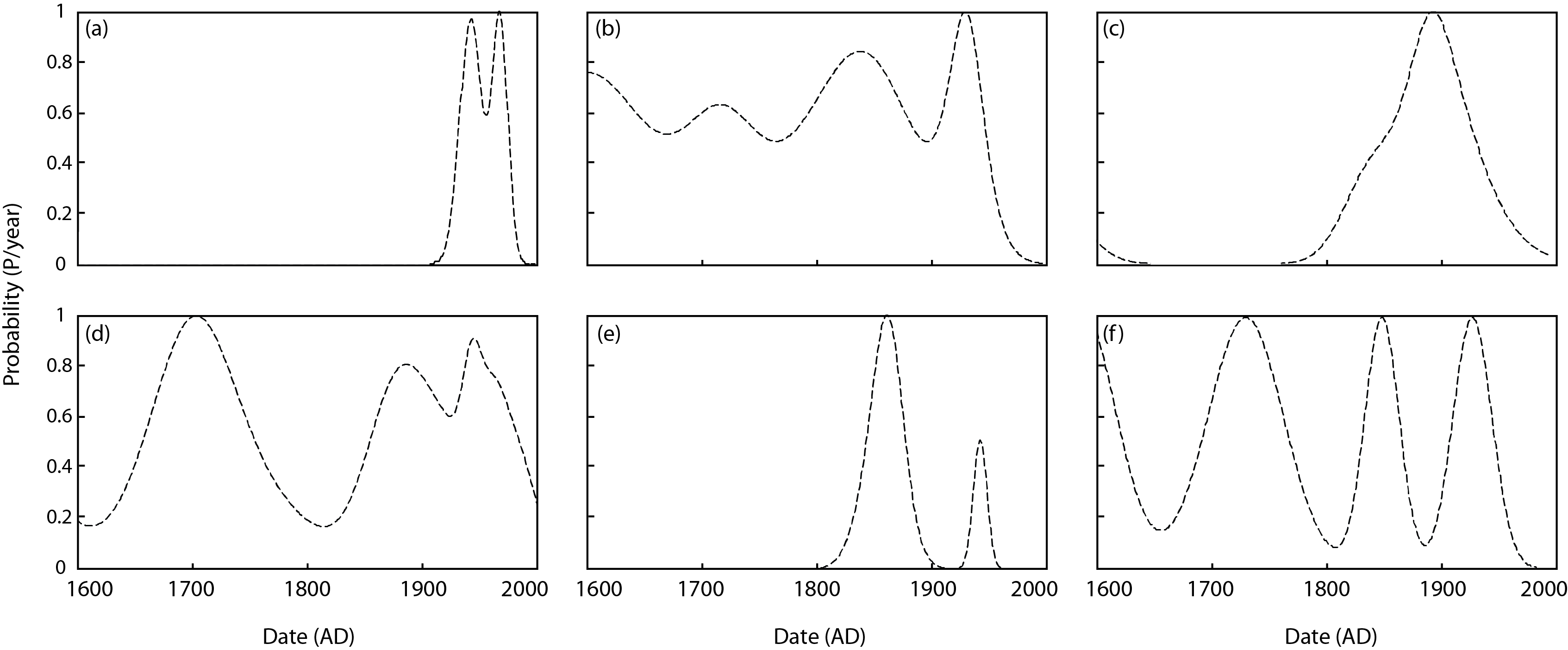


**Figure 12.** PDF of stacked OSL ages taken from six sites (a-f) across the Niobrara Valley Preserve and surrounding ranches, northern Nebraska Sandhills. OSL PDFs have been standardised and ‘non-events’ have been removed from the dataset as part of the pre-processing methods.

1. **ANN2 Inputs**

Historical growing season precipitation and growing season average maximum and minimum temperatures are taken from weather station records kept at Ainsworth Meteorological station, Nebraska (42.58° N, -100.05° W, <10 miles from the study sites) sourced from the National Centres of Environmental Information (https://www.ncdc.noaa.gov/ - accessed on 04/01/2018) from 1908-2014 AD (Figure 13).

**
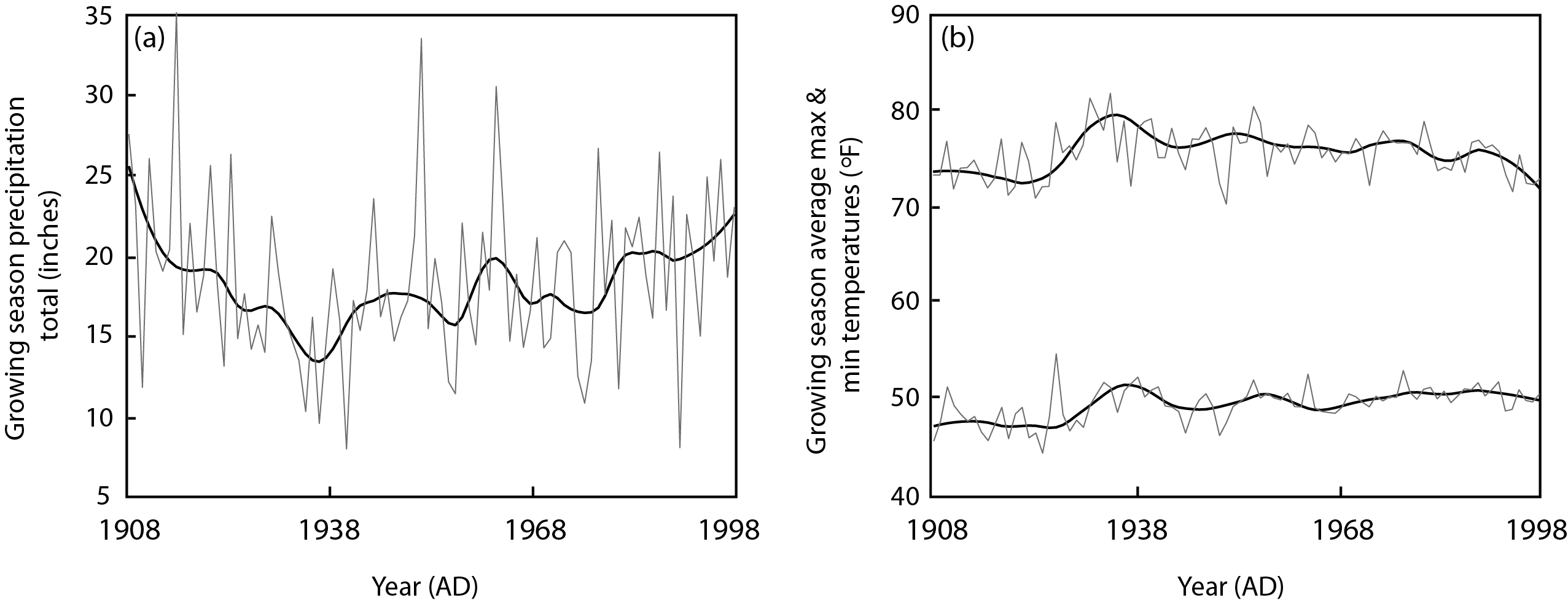
**

**Figure 13.** Historical annual growing season weather data 1908 – 1998 AD: (a) Precipitation totals (inches). Grey line refers to pre-processed growing season annual totals; black line is the post-processed smoothed values that were used by the neural network. (b) Average max and min daily temperatures (°F). Grey line refers to pre-processed annual values; black line is the post-processed smoothed values that were used by the neural network.

**Future climate projections:** A new set of generated annual growing seasons precipitation totals and average maximum and minimum growing seasons temperatures based on noise levels extracted from historical records and three different climate trends.

An autoregressive model was used to identify the historical trends, and thus level of noise found in the growing season precipitation and temperature profiles from 1908 – 1997 AD (Figure 14). Synthetic noise was calculated based on the residual profile of the historical noise relative to the trend from the instrumental climate data. The synthetic noise profile was then added to two combinations of precipitation and temperature data to produce the different climate futures.

**
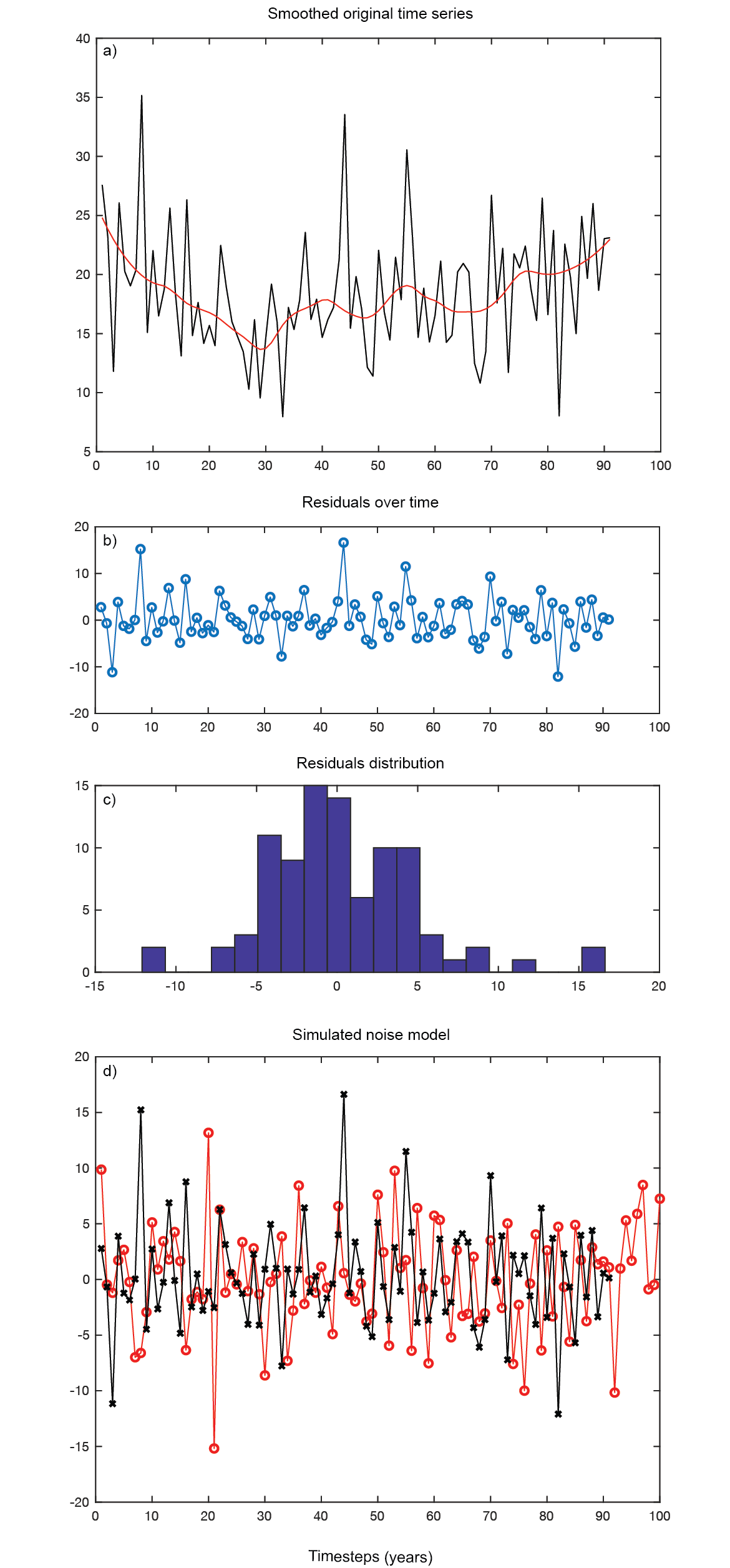
**

**Figure 14.** Example outputs from autoregressive model to identify the level of noise associated with the growing season precipitation dataset and simulate new ‘noise’ for future climates. a) Empirical growing season precipitation measurements with smoothed trend. b) Calculation of annual residuals based on smoothed trend from a). c) Residuals are normally-distributed. d) New simulated noise profile based on the residuals and lags identified in the autoregressive model.

The two climate future trends simulated are detailed in Methods and broadly reflect: Climate 1 – historical long-term averages, and Climate 2 – a gradual increase in temperatures (4.5°F by end of century) to reflect low emissions scenario ^4^. The climatic scenarios do not demonstrate predicted future conditions but are used as proof on concept to demonstrate the capacity of the model under a range of forcing combinations (Figure 15).

**
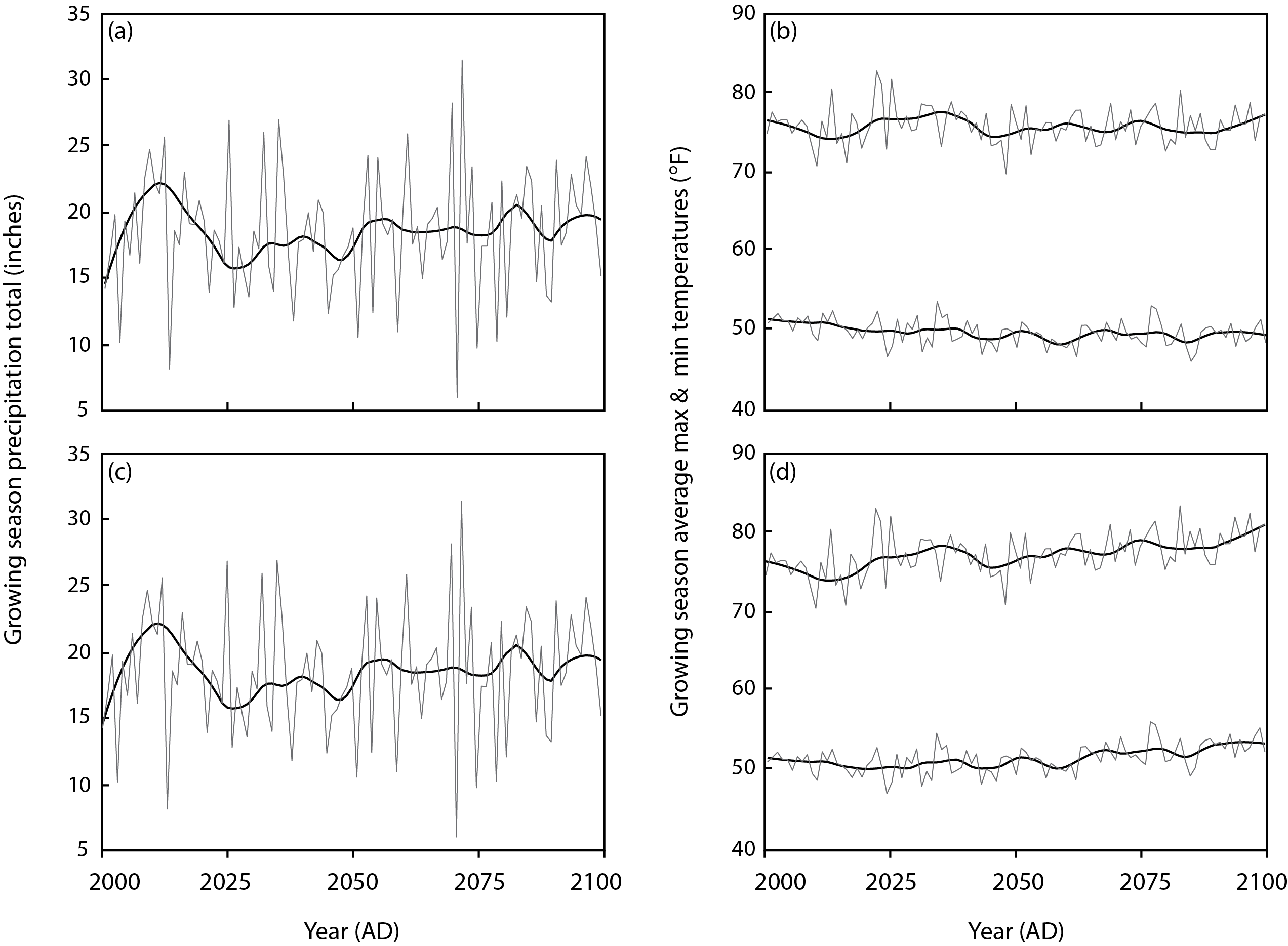
**

**Figure 15.** Growing season precipitation totals and average maximum and minimum growing season daily temperatures associated with the two simulated climate scenarios: Climate 1 (a-b), Climate 2 (c-d).

1. **Processing future scenario outputs**

Final mean and associated standard errors for the predicted output profiles was based on selecting a percentage of the best performing models and their associated output datasets. Reducing the % of accepted models in the dataset improves the accuracy of the final output profile, with only the best performing models used to simulate the predicted likelihood of identifying episodes of deposition. Meanwhile, increasing the number of repeats used in the final mean calculation reduced the overall standard error, increasing the precision of the profile. To improve the standard error, therefore, 5,000 repeats were completed to increase the size of the dataset from which we could subsequently select the top % of model repeats. Figure 16 demonstrates how increasing the number of repeats drastically reduces the standard error associated with the profile, whilst reducing the number of datasets based on model performance improves the resolution of the signal within the dataset; features are not ‘averaged out’. Using an example dataset (Figure 16), results from repeat sensitivity analysis suggested that selecting the top 10% of models from 5,000 repeats provided us with a profile with minimal errors yet was not overly sensitive to noise (e.g. top 5%) (Figure 16). Two distinct identifiable peaks can be seen in all of the 5,000 repeat profiles, suggesting these peaks are a true feature and have not been produced through selective model refinement, with the peaks becoming more pronounced as the model selection becomes more stringent.

**
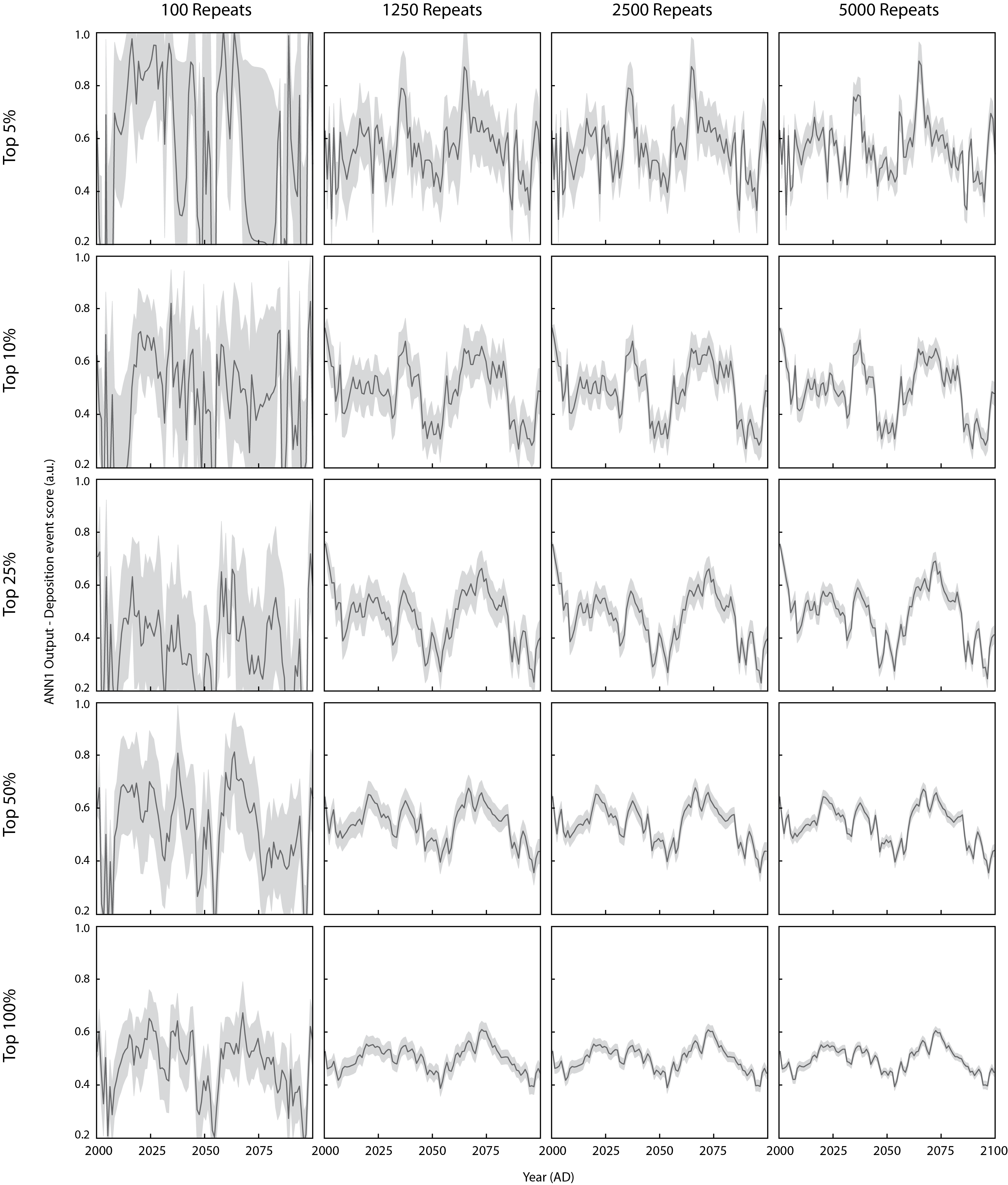
**

**Figure 16.** Example of change in mean and standard error output profile when different numbers of repeats and % of best peak performing models were used in final calculation. Example is based on a hypothetical climate future not used in the main text analysis.

1. **Dominance of climatic signal**

**
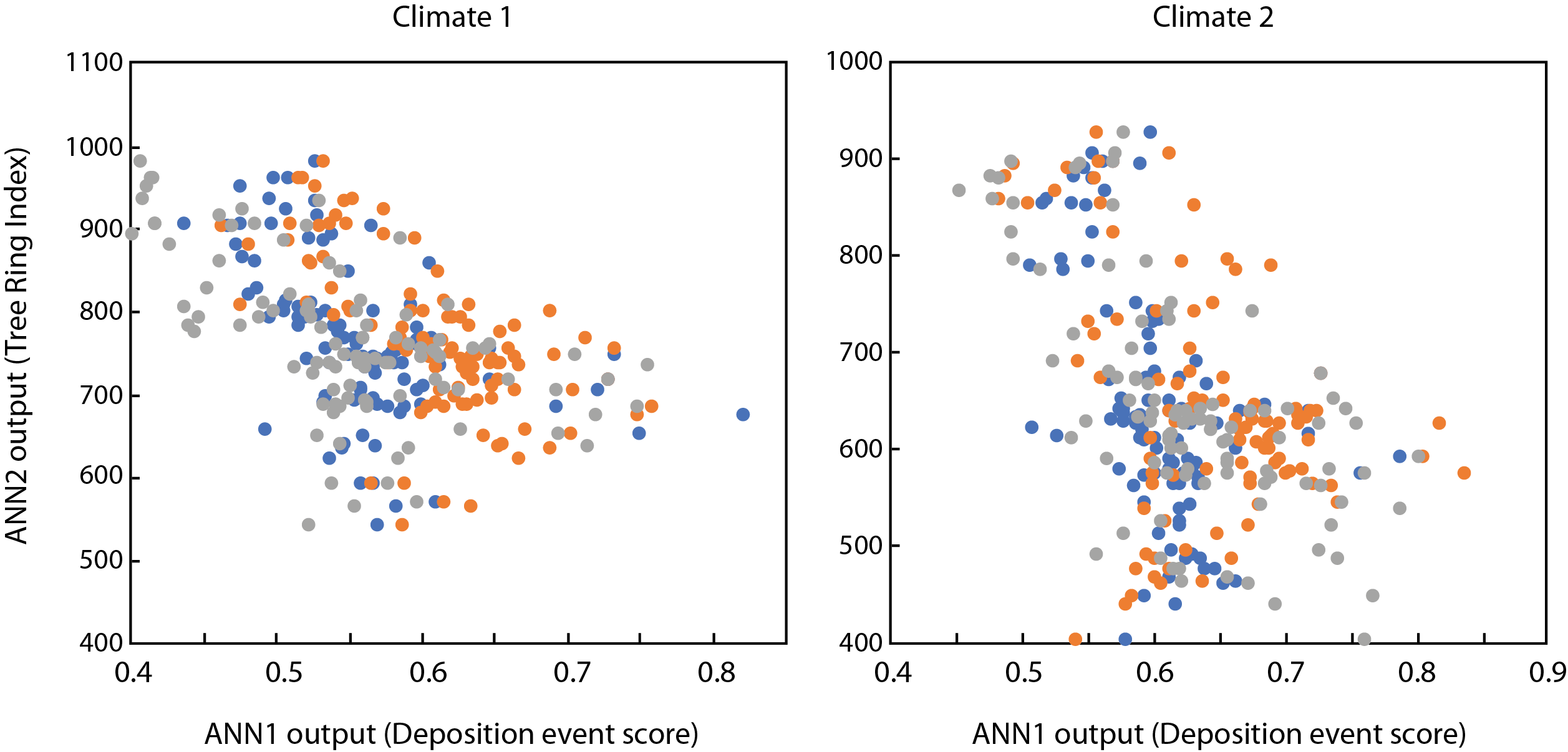
**Results from the scenario forecasting suggest that the majority of the variability found in the deposition event score is explained by the trends shown in the simulated tree ring growth index Include. When the two parameters are plotted against one another, we find a weak anticorrelation associated with vegetation growth (ANN2 output) and the likelihood for periods of sediment deposition to be identified in the record (ANN1 output) (Figure 17).

**Figure 17.** Plots demonstrating the weak anticorrelation associating tree ring growth with the likelihood for episodes of sediment deposition across the two climate profiles simulated. Grey dots: low grazing pressure, orange dots: moderate grazing pressure, blue dots: heavy grazing pressure.

**References**

1. Brown, P. M., Woodhouse, C. A. & Bragg, T. Niobrara Valley Preserve - PIPO - Tree Ring Index - ITRDB NE004. at <https://www.ncdc.noaa.gov/paleo/study/2873>

2. Guyette, R. P., Stambaugh, M. C. & Marschall, J. M. A quantitative analysis of fire history at national parks in the Great Plains. 78 (2011).

3. Bailey, R. M. & Thomas, D. S. G. A quantitative approach to understanding dated dune stratigraphies. *Earth Surf. Process. Landforms* **39,** 614–631 (2014).

4. Bathke, D. J., Oglesby, R. J., Rowe, C. M. & Wilhite, D. A. *Understanding and Assessing Climate Change: Implications for Nebraska*. (2014).
